# Review of the genus *Chrysotimus* Loew from Tibet (Diptera, Dolichopodidae)

**DOI:** 10.3897/zookeys.424.7562

**Published:** 2014-07-08

**Authors:** Mengqing Wang, Hongyin Chen, Ding Yang

**Affiliations:** 1 Key Laboratory of Integrated Pest Management in Crops, Ministry of Agriculture, Institute of Plant Protection, Chinese Academy of Agricultural Sciences, Beijing, 100081, P.R. China; 2 USDA-ARS Sino-American Biological Control Laboratory, Beijing, 100081, P.R. China; 3 Department of Entomology, Chinese Agricultural University, Beijing, 100193, China

**Keywords:** Diptera, Dolichopodidae, *Chrysotimus*, review, new species, Tibet, Taxonomy

## Abstract

A review of the species of the genus *Chrysotimus* from Tibet is provided. The following four species are described as new to science: *C. motuoensis* **sp. n.**, *C. tibetensis* **sp. n.**, *C. xuankuni* **sp. n.**, *C. zhui* **sp. n.** A key to the eight Tibetan species is presented.

## Introduction

The genus *Chrysotimus* Loew, 1857 belongs to the subfamily Peloropeodinae with following characters: often yellow or yellowish hairs and bristles, small first flagellomere, posterior mesonotum distinctly flattened, wing length usually distinctly longer than body length, femora II and III each with strong anterior preapical bristles, most males with hind tarsomere 1 bearing several short black ventral bristles at base, and males with mid tarsomere 1 at least as long as the total of corresponding tarsomeres 2-4, hypopygium with 1-2 epandrial bristles (not processes) for most Chinese species. The genus is distributed worldwide except for the Afrotropical region with 70 known species, of which 14 species are known from the Palaearctic (Negrobov 1978, 1991), and 27 species from the Oriental (Dyte 1975; Yang et al. 2011, Wang et al. 2012). Thirty-seven species are known from China including those newly described herein. *Guzeriplia* Negrobov, 1968, embodies the characters of *Chrysotimus* Loew in the head and thorax with the yellow hairs and bristles and biseriate acr, though it has large hypopygium and a long surstylus and long cercus. For these reasons, it was synonymized with *Chrysotimus* by Yang et al. (2006).

Tibet, together with the Qinghai-Xizang Plateau, Hengduan Mountains, the Himalayas and the Yarlung Zangbo River, is considered to be the one of the most active geological regions and the most sensitive and richest regions in biological diversity in Southeast Asia. Furthermore, southeastern Tibet, bordered by tropical and monsoon rainforest, provides diverse habitats for numerous Oriental insect species, a large number of which are endemic to this area. So, it is likely that more Dolichopodidae and other dipterans will be discovered in the future in this area.

All the specimens in this study were collected from the Oriental part of southeastern Tibet. Four new species are described and a key to the species of *Chrysotimus* from Tibet is presented.

## Materials and Methods

Specimens were studied and illustrated with a ZEISS Stemi 2000–c stereo microscope. Genitalic preparations were made by macerating the apical portion of the abdomen in warm 10% NaOH for 17–20 min. After examination and drawing the hypopygium, it was transferred to 75% alcohol and stored in a microvial pinned below the specimen. All specimens are deposited in the Entomological Museum of China Agricultural University (EMCAU), Beijing, China.

### Abbreviations are as follows

acr acrostichal bristles

ad anterodorsal bristles

av anteroventral bristles

d dorsal bristles

dc dorsocentral bristles

LI fore leg

LII mid leg

LIII hind leg

oc ocellar bristles

pd posterodorsal bristles

pv posteroventral bristles

v ventral bristles

CuAx ratio length of m-cu / length of distal portion of CuA

## Taxonomy

### Genus Chrysotimus

Taxon classificationAnimaliaDipteraDolichopodidae

Loew, 1857

Chrysotimus Loew, 1857: 48. Type species: *Chrysotimus pusio* Loew, 1861, des. Coquillett (1910: 524).
Guzeriplia Negrobov, 1968: 470. Type species: *Guzeriplia chlorina* Negrobov, 1968 (original designation).

#### Key to species (males) of *Chrysotimus* from Tibet

**Table:** 

1	Hypopygium large (about as long as half of abdomen in length)	*Chrysotimus grandis* Wang & Yang
–	Hypopygium small (normal)	2
2	Tarsomere III1 without black ventral bristles at base	*Chrysotimus motuoensis* sp. n.
–	Tarsomere III1 with black ventral bristles at base	3
3	First flagellomere as long as wide (Fig. 5)	4
–	First flagellomere shorter than 2/3 width (Figs 2, 7, 11, 14)	5
4	Nine to ten irregularly paired acr; tarsomere III1 with 2 black ventral bristles at base; cercus long with basal part wide (Fig. 16)	*Chrysotimus zhui* sp. n.
–	Three to four irregularly paired acr; tarsomere III1 with 10-11 black ventral bristles on basal 1/6; cercus short and round in lateral view (Fig. 6)	*Chrysotimus lii* Wang & Yang
5	Acr 3-4 paired; cercus round in lateral view (Figs 8, 15)	6
–	Acr more than 5 pairs; cercus not round in lateral view (Figs 3, 12)	7
6	Tarsomere III1 with more than 10 black ventral bristles on basal 1/6; epandrium with wide lateral process (Fig. 8)	*Chrysotimus linzhiensis* Wang & Yang
–	Tarsomere III1 with 5–6 black ventral bristles at base; epandrium without distinct lateral process (Fig. 15)	*Chrysotimus xuankuni* sp. n.
7	Tarsomere II1 longer than total length of tarsomere II2-5; Tarsomere III1 with 4-6 black ventral bristles at base; cercus long and thick with long bristles (Fig. 12)	*Chrysotimus tibetensis* sp. n.
–	Tarsomere II1 shorter than total length of tarsomere II2-5; Tarsomere III1 with 10–12 black ventral bristles on basal 1/5; cercus short and bifurcated (Fig. 3)	*Chrysotimus bifurcatus* Wang & Yang

### Chrysotimus bifurcatus

Taxon classificationAnimaliaDipteraDolichopodidae

Wang & Yang, 2006

Figs 1–3

Chrysotimus bifurcatus Wang & Yang, 2006. Ent. Fenn. 16: 100. Type locality: China: Tibet, Bomi.

#### Diagnosis.

All coxae yellow. 6-7 irregularly paired acr short and hair-like. Tarsomere III1 with group of 10–12 short black ventral bristles on basal 1/5, and row of 8-9 pv. Cercus bifurcated. For a full description of this species, see Wang and Yang (2006).

**Figures 1–3.**
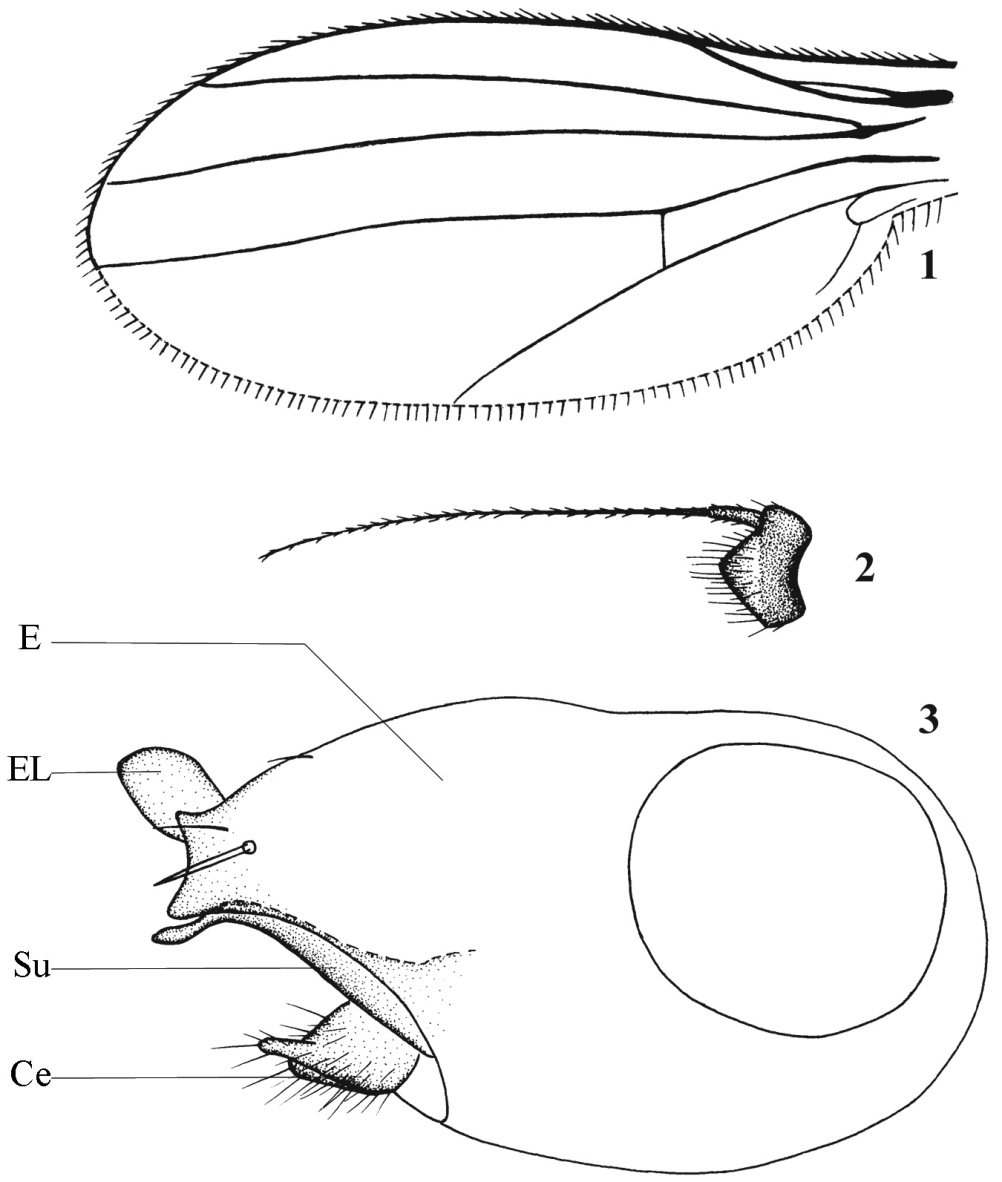
*Chrysotimus bifurcatus* Wang & Yang, 2006, male. **1** wing **2** first flagellomere, lateral view **3** hypopygium, lateral view. **C** cercus; **E** epandrium; **EL** epandrial lobe; **Su** surstylus.

#### Specimens examined.

Type holotype, ♂, Tibet: Bomi, alt. 3050m, 1978.VII.16, leg. Fasheng Li. This specimen was collected from the subtropical rainforest with a sweep net and is deposited in EMCAU.

#### Distribution.

Tibet (Bomi).

### Chrysotimus grandis

Taxon classificationAnimaliaDipteraDolichopodidae

Wang & Yang, 2006

Fig. 4

Chrysotimus grandis Wang & Yang, 2006. Ent. Fenn. 16: 101. Type locality: China: Tibet, Bomi.

#### Diagnosis.

Palpus blackish. 6 strong dc, 6-7 irregularly paired acr short and hair-like. Tarsomere II1 longer than the total length of tarsomeres II2-5. Surstylus with large swollen apex. For a full description of this species, see Wang and Yang (2006).

**Figure 4.**
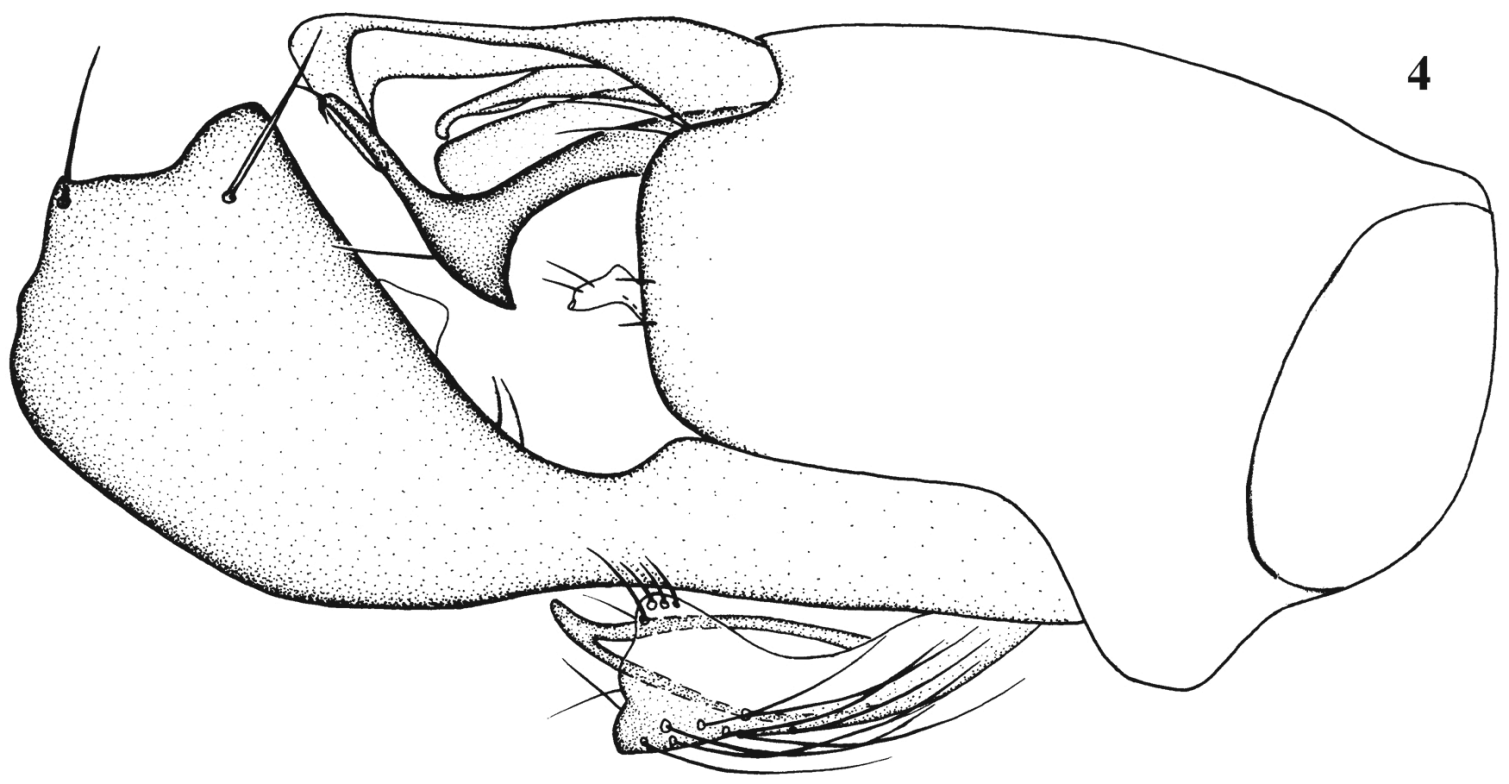
*Chrysotimus grandis* Wang & Yang, 2006, male, hypopygium, lateral view.

#### Specimens examined.

Type holotype, ♂, Tibet: Bomi, alt. 3700m, 1978.VIII.12, leg. Fasheng Li. This specimen was collected from the subtropical rainforest with a sweep net and is deposited in EMCAU.

#### Distribution.

Tibet (Bomi).

### Chrysotimus lii

Taxon classificationAnimaliaDipteraDolichopodidae

Wang & Yang, 2006

Figs 5–6

Chrysotimus lii Wang & Yang, 2006. Ent. Fenn. 16: 102. Type locality: China: Tibet, Bomi.

#### Diagnosis.

First flagellomere subtriangular, as long as wide. Tarsomere III1 with group of 10–11 short black ventral bristles on basal 1/6. For a full description of this species, see Wang and Yang (2006).

**Figures 5–6.**
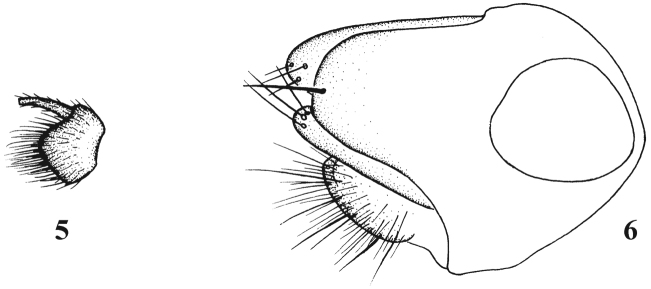
*Chrysotimus lii* Wang & Yang, 2006, male. **5** first flagellomere (arista broken); lateral view **6** hypopygium, lateral view.

#### Specimens examined.

Type holotype, ♂, Tibet: Bomi, alt. 3050m, 1978.VII.16, leg. Fasheng Li. Paratypes, 2 ♂♂, Tibet: Linzhi, 1978. VI.1-3, leg. Fasheng Li. These specimens were collected from the subtropical rainforest with a sweep net and are deposited in EMCAU.

#### Distribution.

Tibet (Bomi, Linzhi).

### Chrysotimus linzhiensis

Taxon classificationAnimaliaDipteraDolichopodidae

Wang & Yang, 2006

Figs 7–8

Chrysotimus linzhiensis Wang & Yang, 2006. Ent. Fenn. 16: 103. Type locality: China: Tibet, Linzhi.

#### Diagnosis.

Palpus pale yellow. First flagellomere short, about 1.5 times wider than long. Tarsomere III1 with group of about 13 short black ventral bristles on basal 1/6. For a full description of this species, see Wang and Yang (2006).

**Figures 7–8.**
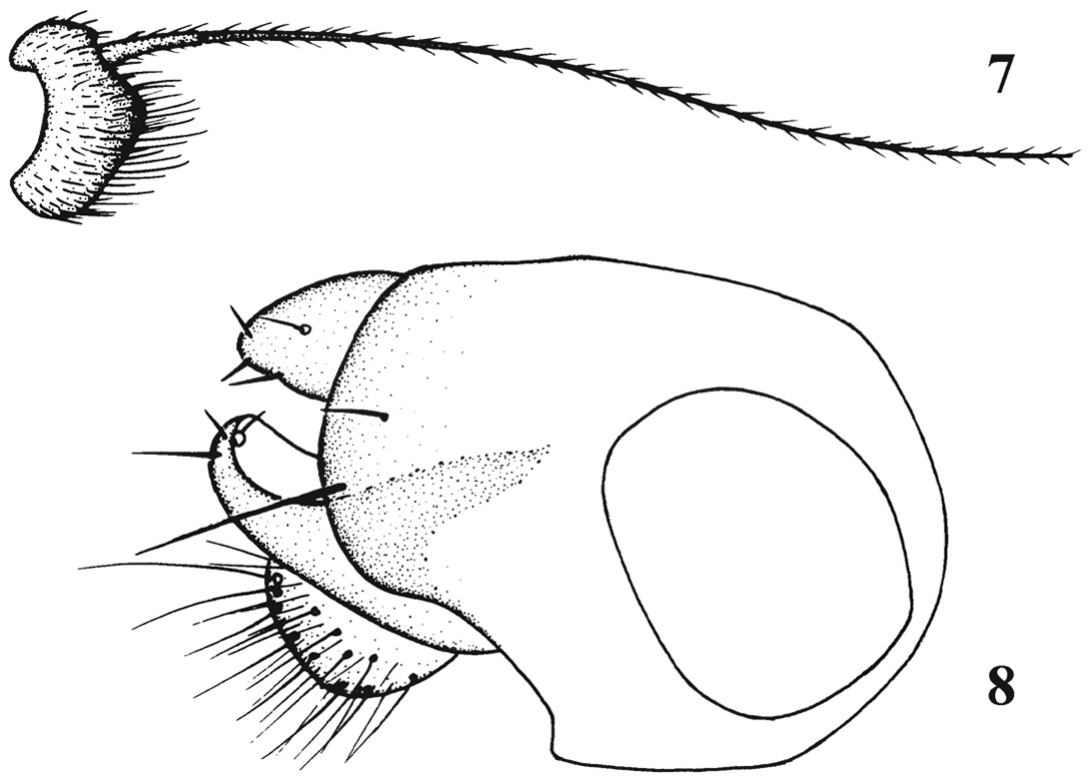
*Chrysotimus linzhiensis* Wang & Yang, 2006, male. **7** first flagellomere, lateral view **8** hypopygium, lateral view.

#### Specimens examined.

Type holotype, ♂, Tibet: Linzhi, alt. 3050m, 1978.VI.1-3, leg. Fasheng Li. This specimen was collected from the monsoon rainforest with a sweep net and is deposited in EMCAU.

#### Distribution.

Tibet (Linzhi).

### Chrysotimus motuoensis sp. n.

Taxon classificationAnimaliaDipteraDolichopodidae

http://zoobank.org/0786482E-B699-45E4-9BC0-065F4A687EAC

Figs 9–10

#### Diagnosis.

Antenna yellow, with both pedicel and 1st flagellomere both with brown dorsal surface; acr absent; abdomen with tergites brilliantly metallic green dorsally and yellow laterally, and with yellow sternites; tarsomere III1 without black ventral spine-like bristles at base.

#### Dexcription.

Male. Body length 1.9 mm, wing length 1.9 mm.

**Figures 9–10.**
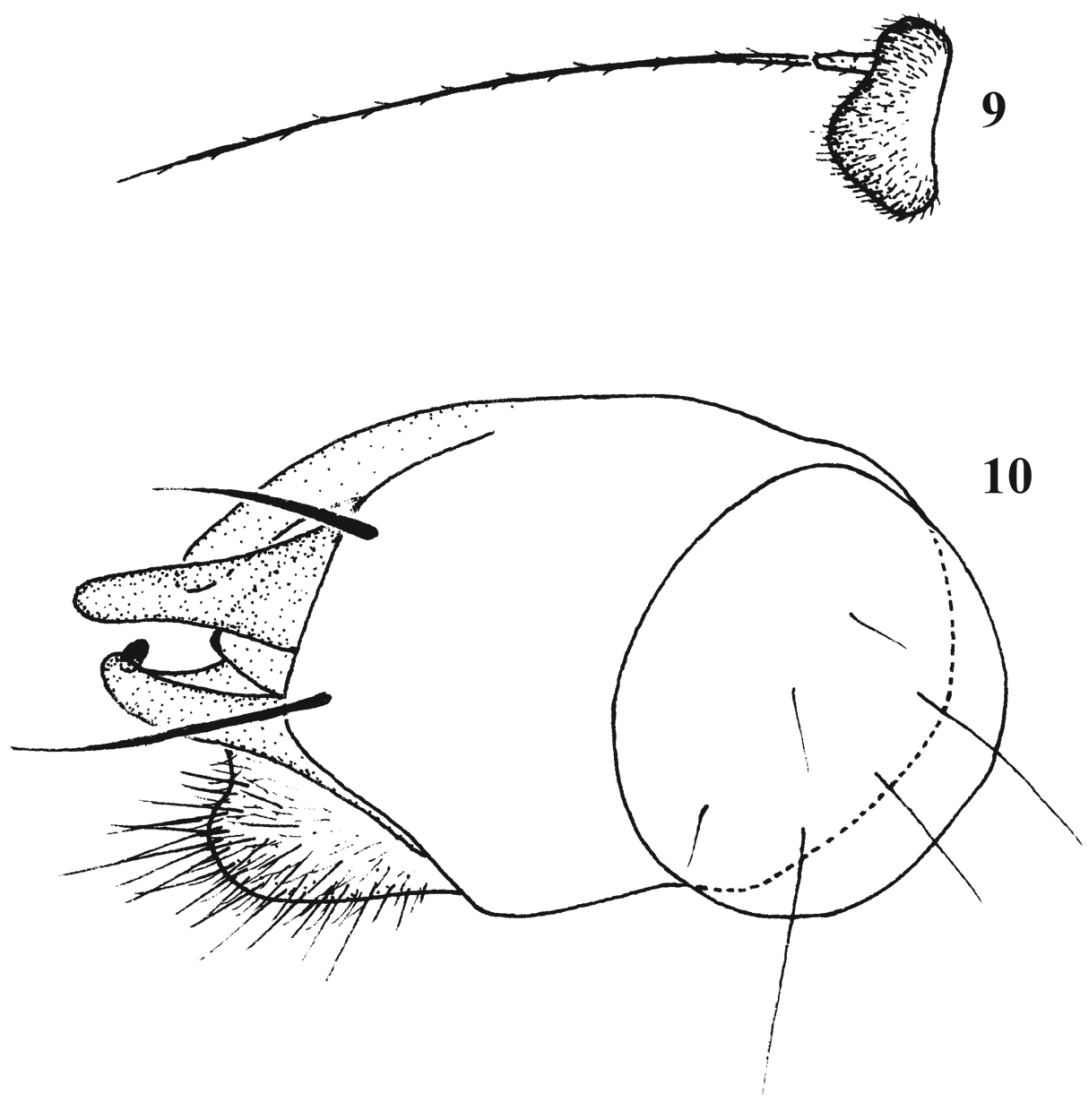
*Chrysotimus motuoensis* sp. n., male. **9** first flagellomere, lateral view **10** hypopygium, lateral view.

**Head** metallic green with gray pollen; frons and face brilliant; eyes separated distinctly; face wide and slightly narrower towards clypeus. Hairs and bristles yellow. Ocellar tubercle weak, with 2 very long oc and 2 very short posterior hairs. Lower postocular bristles (including ventral hairs) pale. Antenna yellow, with both pedicel and 1st flagellomere with brown dorsal surface; first flagellomere (Fig. 9) rather short, about 0.6 times as long as wide; arista apical, with basal segment very short. Proboscis brown, with pale hairs; palpus pale yellow, with pale hairs and 2 brown apical bristles.

**Thorax** metallic green with pale gray pollen, with pleura yellow. Hairs and bristles yellow; 6 dc, acr absent; scutellum with 2 pairs of bristles. Propleuron with 1 pale bristle on lower part. Legs including coxae yellow with 5th tarsomeres brown. Hairs and bristles on legs pale yellow; coxa I with 3-4 anterior and apical bristles, coxa II with 2 anterior and apical bristles, coxa III with 1 brown outer bristle near middle. Femora II and III each with 1 apical av. Tibia II with 2 ad and 2 pd, apically with 3 bristles; tibia III with 1 ad and 2 pd, apically with 3 bristles. All tarsomere 1 each with row of v. Tarsomere III 1 without black ventral spine-like bristles at base. Relative lengths of tibia and 5 tarsomeres of legs. LI 3.4 : 2.2 : 1.0 : 0.8 : 0.6 : 0.4; LII 4.0 : 2.4 : 1.1 : 0.9 : 0.5 : 0.4; LIII 4.7 : 1.0 : 1.4 : 0.9 : 0.6 : 0.4.

Wing hyaline; veins brownish, R_4+5_ and M parallel apically; CuAx ratio 0.3. Squama brown with brown hairs. Halter pale yellow.

Abdomen metallic green with pale gray pollen, tergites brilliant, sternites and lateral portion yellow. Hairs and bristles on tergites dark brown, and pale yellow on sternites.

Hypopygium (Fig. 10): Epandrium with truncate apex bearing 2 epandrial bristles, apically with wide lateral epandrial process; long surstylus with inner spine-like process; cercus somewhat round, with moderately long hairs; hypandrium with round apex.

Female. Body length 1.8 mm, wing length 1.7 mm. Similar to male, but antenna entirely yellow.

#### Specimens examined.

Holotype ♂, Tibet: Motuo county, alt. 1100m, 2012.VIII.26, leg. Xuankun Li. Paratype, 1 ♀ same data as holotype. These specimens were collected from the subtropical rainforest with a sweep net and are deposited in EMCAU.

#### Distribution.

Known only from the type locality in Tibet.

#### Remarks.

This new species is similar to *Chrysotimus guangxiensis* Yang & Saigusa, but may be separated from the latter by brown proboscis, pale yellow palpus, and the tergites metallic green, sternites and lateral portion yellow. In *guangxiensis*, it has yellow proboscis, brown palpus, and whole abdomen metallic green (Yang and Saigusa 2001).

#### Etymology.

The specific epithet derives from the type locality Motuo (Tibet).

### Chrysotimus tibetensis sp. n.

Taxon classificationAnimaliaDipteraDolichopodidae

http://zoobank.org/17995E26-9DAB-4CD9-AE81-1DC2068681FD

Figs 11–13

#### Diagnosis.

Antenna whole brown; acr 5–6 irregular pairs; abdomen whole brilliant metallic green; tarsomere III1 with 4–6 short black ventral bristles at base; cercus long and thick, with sparse hairs and long bristles.

#### Description.

Male. Body length 1.7–1.8 mm, wing length 2.2–2.5 mm.

**Figures 11–13.**
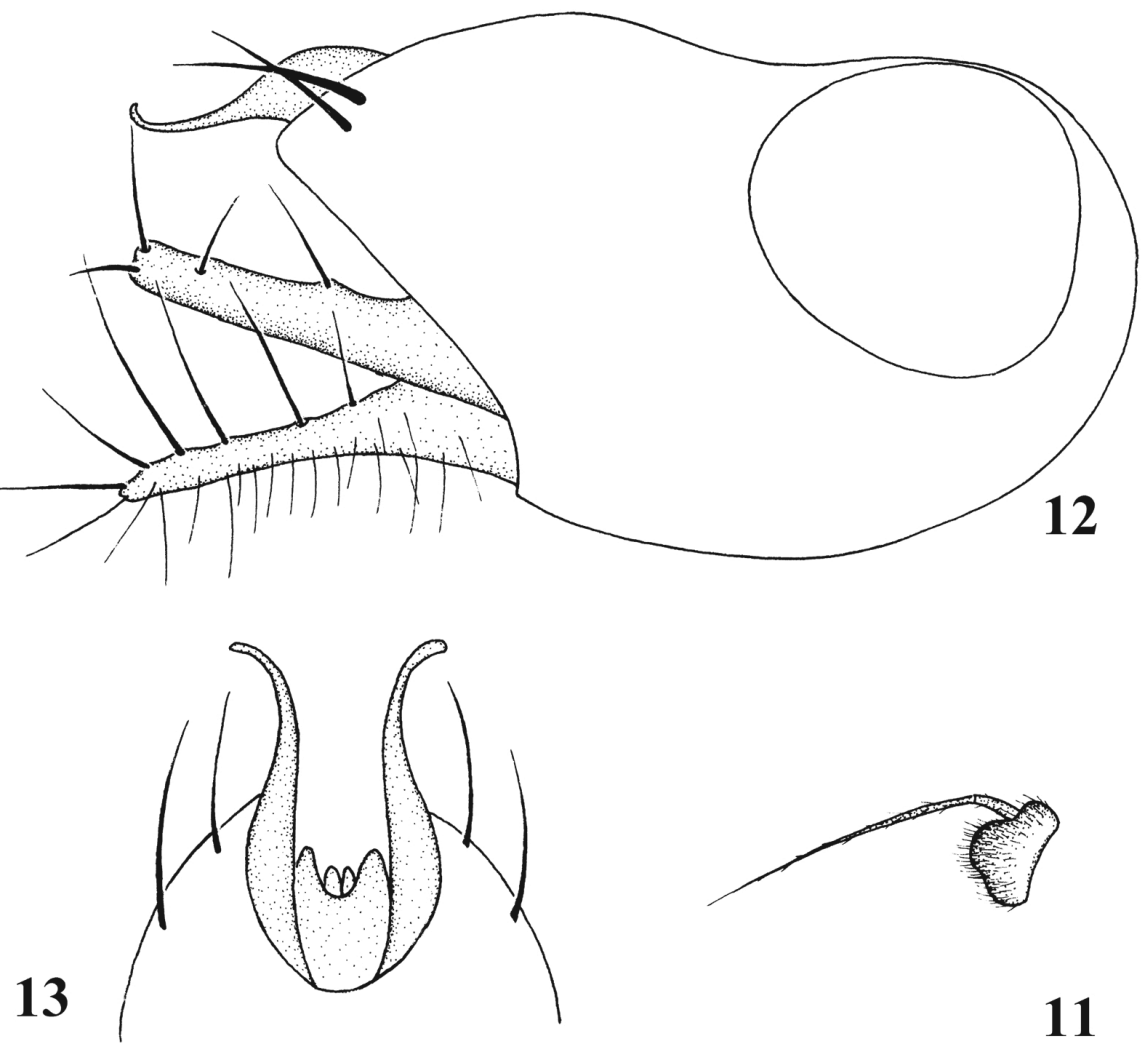
*Chrysotimus tibetensis* sp. n., male. **11** first flagellomere, lateral view **12** genitalia, lateral view **13** tip of hypopygium, ventral view.

Head metallic green with gray pollen; frons and face brilliant; eyes separated distinctly. Hairs and bristles on head yellow. Ocellar tubercle weak, with 2 very long oc and 2 very short posterior hairs. Lower postocular bristles (including ventral hairs) pale. Antenna (Fig. 11) brown; first flagellomere with round apex, rather short, about 0.6 times as long as wide; arista dorsal, with basal segment very short. Proboscis brown, with pale hairs; palpus pale yellow, with pale hairs and 2 brown apical bristles.

Thorax metallic green with pale gray pollen, mesonotum and scutellum brilliant. Hairs and bristles on thorax yellow; 6 dc, 5–6 irregular paired acr; scutellum with 2 pairs of bristles. Propleuron with 1 pale bristle on lower portion. Legs including coxae yellow with 5th tarsomeres brown (some specimens with black legs, except for yellow femoral base and tip). Hairs and bristles on legs pale yellow; coxa I with 2-4 anterior and apical bristles, coxa II with 3-4 anterior and apical bristles, coxa III with 1 brown outer bristle near middle. Femur II with 1 av and 1 pv apically, femur III with 1 av apically. Tibia II with 2 ad and 1 pd, apically with 3 bristles; tibia III with 2 ad, 2 pd, and row of pv, apically with 3 bristles. All tarsomere 1 each with row of v. Tarsomere III1 with 4-6 short black ventral bristles at base. Relative lengths of tibia and 5 tarsomeres of legs. LI 4.2 : 2.4 : 1.2 : 1.0 : 0.6 : 0.4; LII 5.6 : 3.2 : 1.2 : 0.8 : 0.5 : 0.4; LIII 6.4 : 2.8 : 1.6 : 1.2 : 0.8 : 0.6.

Wing hyaline; veins brownish, R_4+5_ and M parallel apically; CuAx ratio 0.27. Squama yellow with pale hairs. Halter pale yellow.

Abdomen metallic green with pale gray pollen, tergites and sternites brilliant. Hairs and bristles on tergites dark brown.

Hypopygium (Figs 12–13): Epandrium with acute apex, apically with 2 epandrial bristles and wide lateral epandrial process, bearing thin and curved apex; long and thick surstylus with long bristles; cercus long and thick, with sparse hairs and long bristles; hypandrium shorter than epandrium.

Female. Body length 1.5–1.7 mm, wing length 1.9–2.0 mm. Similar to male, with whole abdomen metallic green.

#### Specimens examined.

Holotype ♂, Tibet: Linzhi, 2012. IX.2–12 (M). Paratypes, 32♂♂ 14♀♀, same data as holotype. Other specimens: 1♂4♀♀, Tibet: Linzhi, 2012.VIII (M); 3♂32♀♀, Tibet: Linzhi, 2012. IX.22–X.1 (M); 12♂♂7♀♀, Tibet: Linzhi Sejila Mountain, alt. 3810m, 2012.VIII.25–IX.2 (M); 4♂♂11♀♀, Tibet: Linzhi Sejila Mountain, alt. 3260m, 2012.VIII.12–18 (M); 3♂♂2♀♀, Tibet: Linzhi Sejila Mountain Lulangdong, alt. 3349m, 2012.VIII.25–IX. 2 (M); 7♂♂15♀♀, Tibet: Linzhi Sejila Mountain Lulangdong, alt. 3312m, 2012.VIII.18–25 (M); 8♂♂, Tibet: Linzhi Sejila Mountain Kouxi, alt. 3780m, 2012.VIII.15–18 (M); 18♂♂13♀♀, same site, 2012.VIII.19–25 (M); 5♂♂11♀♀, Tibet: Linzhi Nongmuxueyuan Dianzhan, alt. 3573m, 2012.VII.7–15 (M), all leg. Chaodong Zhu. These specimens were collected from the monsoon rainforest with Malaise traps and are deposited in EMCAU.

#### Distribution.

Known only from the type locality in Tibet.

#### Remarks.

This new species is similar to *Chrysotimus ningxianus* Wang, Yang & Grootaert, but may be separated from the latter by rounded first flagellomere, and the epandrium with 1 lateral epandrial process. In *ningxianus*, it has triangular first flagellomere, and the epandrium has 2 lateral epandrial processes (Wang et al. 2005).

#### Etymology.

The specific epithet derives from the type locality in Tibet.

### Chrysotimus xuankuni sp. n.

Taxon classificationAnimaliaDipteraDolichopodidae

http://zoobank.org/53EFFF74-0CF8-46D2-B9DE-5BC83BB27D7D

Figs 14–15

#### Diagnosis.

Antenna blackish; first flagellomere rather short, about 0.4 times as long as wide; ac 3–4 irregular pairs; tibia I with row of 8–9 d; abdomen whole brilliant metallic green; tarsomere III1 with 5–6 short black ventral bristles at base; epandrium without distinct lateral process; cercus round, with moderate hairs.

#### Description.

Male. Body length 1.5 mm, Wing length 1.6 mm.

**Figures 14–15.**
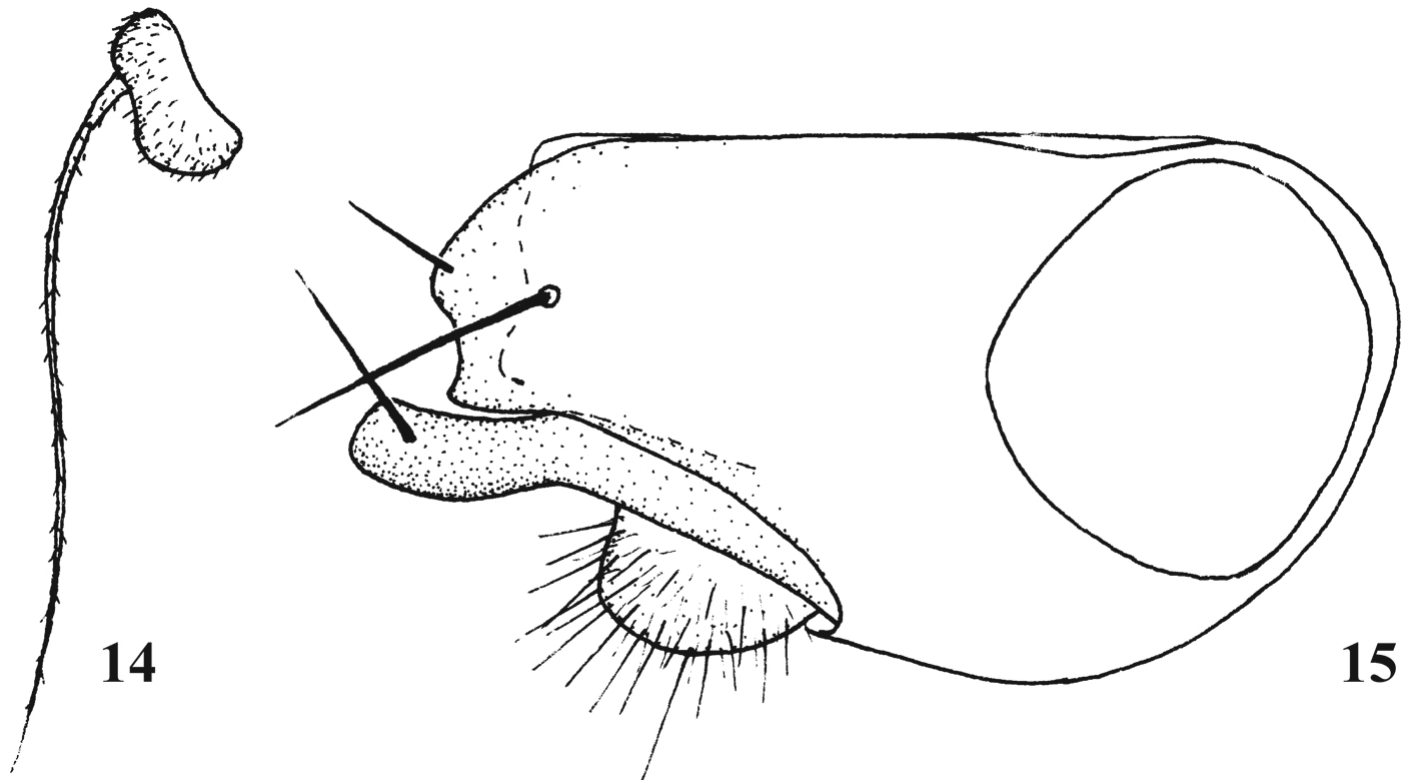
*Chrysotimus xuankuni* sp. n., male. **14** first flagellomere, lateral view **15** genitalia, lateral view.

Head metallic green with gray pollen; frons and face brilliant; eyes separated distinctly. Hairs and bristles on head yellow. Ocellar tubercle weak, with 2 very long oc and 2 very short posterior hairs. Lower postocular bristles (including ventral hairs) pale. Antenna blackish; first flagellomere (Fig. 14) rather short, about 0.4 times as long as wide; arista dorsal, with basal segment very short. Proboscis blackish, with brown hairs; palpus brown, with brown hairs and 2 brown apical bristles.

Thorax metallic green with pale gray pollen, mesonotum and scutellum brilliant. Hairs and bristles on thorax yellow; 6 dc, 3-4 irregular paired acr; scutellum with 2 pairs of bristles. Propleuron with 1 pale bristle on lower portion. Legs including coxae yellow with 5th tarsomeres brown. Hairs and bristles on legs pale yellow; coxa I with 3–4 anterior and apical bristles, coxa II with 3–4 anterior and apical bristles, coxa III with 1 brown outer bristle near middle. Femora II and III each with 1 av apically. Tibia I with row of 8–9 d; tibia II with 2 ad and 2 pd, apically with 3 bristles; tibia III with 1 ad, 2 pd, apically with 3 bristles. All tarsomere 1 each with row of v. Tarsomere III1 with 5–6 short black ventral bristles at base. Relative lengths of tibia and 5 tarsomeres of legs. LI 4.2 : 2.0 : 1.0 : 0.8 : 0.6 : 0.6; LII 5.2 : 2.4 : 1.6 : 1.2 : 0.6 : 0.6; LIII 5.2 : 1.4 : 1.4 : 0.8 : 0.6 : 0.6.

Wing hyaline; veins brownish, R_4+5_ and M parallel apically; CuAx ratio 0.28. Squama yellow with pale hairs. Halter brownish.

Abdomen metallic green with pale gray pollen, tergites and sternites brilliant. Hairs and bristles on abdomen dorsal dark brown.

Hypopygium (Fig. 15): Epandrium with wide apex, apically with invision, bearing 2 epandrial bristles, but no distinct lateral epandrial process; long and thick surstylus with curved apex; cercus round, with moderate hairs; hypandrium shorter than epandrium.

#### Female.

Body length 1.5–1.6 mm, Wing length 1.5–1.6 mm. Similar to male, with whole abdomen metallic green.

#### Specimens examined.

Holotype ♂, Tibet: Motuo county, alt. 1100m, 2012. VII. 26, leg. Xuankun Li. Paratype, 2♀♀, same data as holotype. These specimens were collected from the subtropical rainforest with a sweep net and are deposited in EMCAU.

#### Distribution.

Known only from the type locality in Tibet.

#### Remarks.

This new species is similar to *Chrysotimus guangdongensis* Wang, Yang & Grootaert, but may be separated from the latter by the rowed d on tibia I, and the epandrium bearing no distinct lateral process. In *guangdongensis*, it lacks distinct rowed d on tibia I, and features a lateral process of the epandrium (Wang et al. 2005).

#### Etymology.

The specific epithet derives from the collector of type species Xuankun Li.

### Chrysotimus zhui sp. n.

Taxon classificationAnimaliaDipteraDolichopodidae

http://zoobank.org/0F6879D6-3630-4423-B4B0-D77A4D7ED7DD

Fig. 16

#### Diagnosis.

Antenna brown; first flagellomere subtriangular, about as long as wide; acr 9–10 irregular pairs; tarsomere III1 with 2 short black ventral bristles on long kidney-shaped black spot; abdominal dorsum brilliant metallic green; epandrium with long and curved surstylus; cercus long with wide basal part, with long hairs and bristles.

#### Description.

Male. Body length 1.9–2.0 mm, Wing length 2.2–2.4 mm.

Head metallic green with gray pollen; frons and face brilliant; eyes separated distinctly. Hairs and bristles on head yellow. Ocellar tubercle weak, with 2 very long oc and 2 very short posterior hairs. Lower postocular bristles (including ventral hairs) pale. Antenna brown; first flagellomere subtriangular, about as long as wide; arista dorsal, with basal segment very short. Proboscis blackish, with brown hairs; palpus yellow, with yellow hairs and 2 brownish apical bristles.

Thorax metallic green with pale gray pollen, mesonotum and scutellum brilliant. Hairs and bristles on thorax yellow; 6 dc, 9–10 irregular paired acr; scutellum with 2 pairs of bristles. Propleuron with 1 pale bristle on lower portion. Legs including coxae yellow with 5th tarsomeres brown. Hairs and bristles on legs pale yellow; coxa I with 3–4 anterior and apical bristles, coxa II with 4–6 anterior and apical bristles, coxa III with 1 brown outer bristle near middle. Femur II with 1 av and 1 pv apically, femur III with 1 av apically. Tibia II with 2 ad and 2 pd, apically with 3 bristles; tibia III with 2 ad, 2 pd, and row of pv, apically with 3 bristles. All tarsomere 1 each with row of v. Tarsomere III1 with long kidney-shaped black spot at base, with 2 short black ventral bristles at black spot. Relative lengths of tibia and 5 tarsomeres of legs. LI 4.4 : 2.8 : 1.0 : 0.8 : 0.5 : 0.5; LII 6.0 : 3.6 : 1.4 : 1.0 : 0.6 : 0.6; LIII 6.4 : 2.0 : 1.4 : 1.0 : 0.7 : 0.6.

Wing hyaline; veins brownish, R_4+5_ and M parallel apically; CuAx ratio 0.25. Squama yellow with pale hairs. Halter pale yellow.

Abdomen metallic green with pale gray pollen, tergites brilliant. Hairs and bristles on abdomen dorsal dark brown.

**Figure 16.**
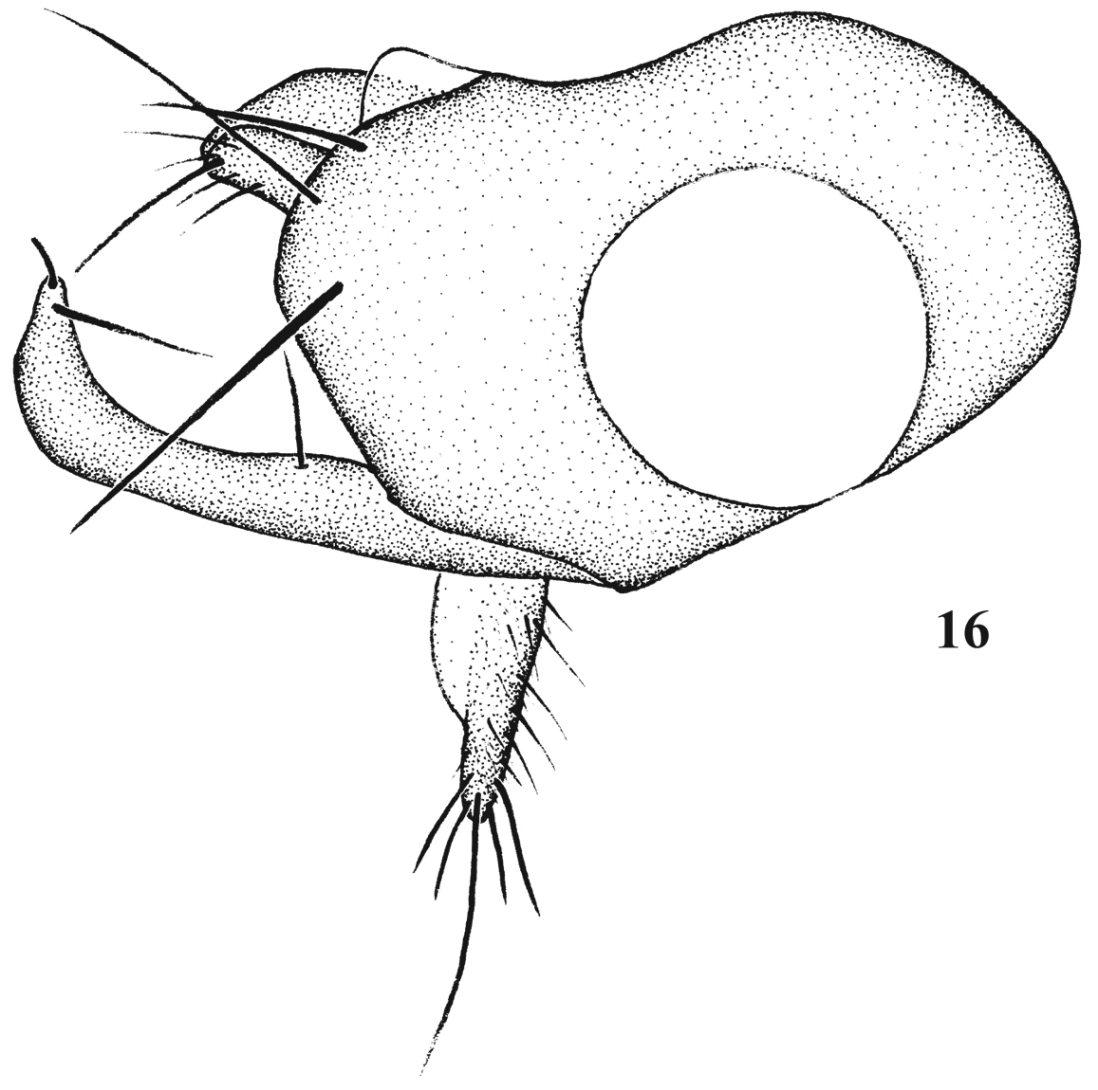
*Chrysotimus zhui* sp. n., male. hypopygium, lateral view.

Hypopygium (Fig. 16) metallic green (except pale hypandrium): Epandrium distinctly longer than wide, apically with 3 epandrial bristles and round short finger-like lateral epandrial process; long and curved surstylus with long bristles; cercus long with wide basal part, with long hairs and bristles; hypandrium short, pale.

#### Female.

Body length 2.0 mm, Wing length 2.8 mm. Similar to male, with whole abdomen metallic green.

#### Specimens examined.

Holotype ♂, Tibet: Linzhi Sejila Mountain Kouxi, alt. 3780m, 2012. VIII. 15–18 (M), leg. Chaodong Zhu; Paratype, 1♂ 1♀, same data as holotype. These specimens were collected from the monsoon rainforest with a Malaise trap and are deposited in EMCAU.

#### Distribution.

Known only from the type locality in Tibet.

#### Remarks.

This new species is similar to *Chrysotimus ningxianus* Wang, Yang & Grootaert, but may be separated from the latter by the black spot at tarsomere III1 base, and the epandrium with 1 single finger-like lateral epandrial process. In *ningxianus*, it has no black spot at tarsomere III1 base, and the epandrium has 2 lateral epandrial processes (Wang et al. 2005).

#### Etymology.

The specific epithet derives from the collector of types Dr. Chaodong Zhu (Beijing).

## Supplementary Material

XML Treatment for Chrysotimus

XML Treatment for Chrysotimus bifurcatus

XML Treatment for Chrysotimus grandis

XML Treatment for Chrysotimus lii

XML Treatment for Chrysotimus linzhiensis

XML Treatment for Chrysotimus motuoensis

XML Treatment for Chrysotimus tibetensis

XML Treatment for Chrysotimus xuankuni

XML Treatment for Chrysotimus zhui
